# Collaborative Routing Optimization Model for Reverse Logistics of Construction and Demolition Waste from Sustainable Perspective

**DOI:** 10.3390/ijerph19127366

**Published:** 2022-06-16

**Authors:** Qianqian Chen, Wenzhu Liao

**Affiliations:** School of Management Science and Real Estate, Chongqing University, Chongqing 400045, China; cqq@cqu.edu.cn

**Keywords:** construction and demolition waste, recycling, sustainable, reverse logistics, route

## Abstract

The construction industry is developing rapidly along with the acceleration of urbanization but accompanied by an increased amount of construction and demolition waste (CDW). From the perspective of sustainability, the existing research has mainly focused on CDW treatment or landfill disposal, but the challenge of reverse logistics of CDW recycling that provides overall CDW route planning for multiple participants and coordinates the transportation process between multiple participants is still unclear. This paper develops an optimization model for multi-depot vehicle routing problems with time windows (MDVRPTW) for CDW transportation that is capable of coordinating involved CDW participants and suggesting a cost-effective, environment-friendly, and resource-saving transportation plan. Firstly, economic cost, environmental pollution, and social impact are discussed to establish this optimization-oriented decision model for MDVRPTW. Then, a method combined with a large neighborhood search algorithm and a local search algorithm is developed to plan the transportation route for CDW reverse logistics process. With the numerical experiments, the computational results illustrate the better performance of this proposed method than those traditional methods such as adaptive large neighborhood search algorithm or adaptive genetic algorithm. Finally, a sensitivity analysis considering time window, vehicle capacity, and carbon tax rate is conducted respectively, which provides management implications to support the decision-making of resource utilization maximization for enterprises and carbon emission management for the government.

## 1. Introduction

Construction and demolition waste (CDW) is a major component of urban solid waste, usually generated in the process of demolition, construction, renovation, and maintenance of buildings [1]. It’s estimated by The United Nations that 68% of the world population will live in urban areas by 2050. With increasing urbanization, the vigorous development of the construction industry and the improvement of community living standards bring a rapid acceleration of the generation of CDW [2]. In recent years, as a large number of projects have been implemented, such as large-scale infrastructure construction and transformation and city comprehensive management, China has become the country with the largest output of CDW in the world. According to the data from the Ministry of Housing and Urban-Rural Development of China, the annual output of city construction waste is over 2 billion tons in 2021, which is about 10 times the amount of domestic waste generated, accounting for 40% of the total urban solid waste. It is estimated by EPA that 600 million tons of CDW were generated in the United States in 2018. However, the recycling rate of CDW in China is very low, approximately 3% to 10% [3], while US is around 76% [4]. According to Eurostat, the recycling rate considering non-hazardous CDW ranges from 24% to 100% in the EU-28, with 73% in France, 98% in UK, and 100% in Netherlands, Ireland, Malta and North Macedonia. Since the growth rate of CDW utilization is lagging behind that of CDW generation, city managers have been seeking to increase the efficacy of CDW management methods in recent years.

The CDW management is a vital part of the government’s strategy to achieve sustainable development [5], which includes the collection, sorting, transportation, processing, treatment, and disposal in an organized manner. The 3R principle (i.e., reduce, reuse, and recycle), is playing a significant role in CDW management, as the most commonly used universal principle [6]. When it comes to the implementation of a waste reduction strategy, it is vital for stakeholders in the construction industry to communicate and cooperate properly and reach an agreement on the understanding of 3R [7]. After that, it is of great importance to sort, remove or crush CDW through special equipment and select reusable resources. Most CDW can be reused after demolition works [7]. Implementing on-site CWD separation is one of the most important CDW recycling methods, which promotes materials recovery and reduces waste disposal at landfill sites [8]. At present, China advocates on-site classified disposal of CDW, which can be divided into metal, inorganic non-metal, and other categories. No matter how efficient the reduce and reuse strategies are, the recycling strategy is an inevitable strategy to reprocess the waste into products or materials in CDW management. The recycling of CDW can achieve greater social, economic, and environmental effects by conserving resources, reducing pollution, and stimulating the economy [9]. The non-recyclable CDW is finally sent to the incineration plant or landfill.

Reverse logistics is a process to recycle used, outdated or damaged products from customers to final treatment. As CDW is viewed as a valuable resource because many types of construction waste can be reused or recycled, such as concrete, metal, brick, wood, paper, glass, and rubber. Reverse logistics of CDW management is viewed as one of the essential components to move toward sustainability [10]. On the one hand, efficient recycling of CDW from generation sites to final disposal centers could reduce production costs by saving natural resources. On the other hand, it could reduce CO_2_ emissions and landfilled waste mitigation. However, the existing waste management suffers from high costs of transportation [11]. Managers are eager to choose the optimal transportation plans, as an optimal waste transportation plan can effectively reduce the cost of waste transportation [12,13]. An optimal vehicle route planning can effectively bring travel cost reduction and ultimately improve waste resource efficiency [14]. Hence, how to optimize the reverse logistics and find a transportation plan for sustainable operations which considers economic, environmental, and social benefits has arisen research attention [15,16].

This paper discusses a vehicle routing problem for reverse logistics of CDW from the perspective of economic, environmental, and social impact, and provides a cost-effective, environment-friendly, and resource-saving transportation scheme suitable for multiple participants involved from CDW generation to transfer station. This could assist owners in the decision-making of resource utilization maximization and carbon emission management in the whole transportation process of CDW management. Therefore, the main contributions of this paper are presented in the following aspects. First, we establish a collaborative optimization model for multi-depot vehicle routing problems with time windows for recycling construction and demolition waste from the perspective of the economy, environment, and society. Next, we suggest a cost-effective, environment-friendly, and resource-saving transportation plan with a hybrid adaptive neighborhood search algorithm. After that, we discuss the computational results concerning performance analysis, time window analysis, vehicle capacity analysis, and carbon tax rate analysis with the numerical experiments. Finally, several useful management implications are given to support the decision-making of resource utilization maximization for enterprises and carbon emission management for the government.

## 2. Literature Review

Recycling CDW can lead to significant reductions in emissions, energy use, and global warming potential, and conserves landfill space when compared to the disposal of wastes in landfills [17]. Reverse logistics has attracted the attention of a large number of scholars as it can improve the efficiency of recycling. Therefore, CDW transportation is quite a relevant segment of the entire CDW management system, and it is of great importance to find suitable transportation routes. Three parts of related literature are discussed in this section: sustainability in the construction industry, reverse logistics, and vehicle routing problems.

### 2.1. Sustainability in Construction Industry

The necessity of sustainable development has become a topic widely recognized by the international community. In 2015, the United Nations formulated the 2030 Agenda for Sustainable Development, which set out the Sustainable Development Goals (SDGs), comprising 17 global goals gathered from the economic, environmental, and social dimensions. In addition, the circular economy is an essential measure for achieving sustainable development, which aims to reduce resource input and promote the efficient use of resources.

With the increasing concern on large resource consumption in the construction industry, sustainable development has received growing attention among various parties. Jin et al. [18] designed an empirical study on the current practice and trend of CDW management in China and provide directions on the sustainable treatment of CDW in developing countries. A literature review was conducted by Ghisellini et al. [19] to assess if the circular economy approach is environmentally and economically sustainable for CDW management. The result showed that, in most cases, the recycling of CDW at the end-of-life of a building as well as the production of recycled products can contribute to sustainable development. Hossain et al. [20] identified the implications, considerations, contributions, and challenges of circular economy in the construction industry, which might promote effective implementation of the circular economy into the industry for promoting sustainable construction. It is integral to establish a detailed relationship between the SDGs, CDW management, and the circular economy [21]. These linkages can improve competitiveness, stimulate innovation, and boost economic growth.

In recent years, Life Cycle Assessment (LCA) has gained increasing attention in the building industry as it is an efficient tool to investigate sustainability. LCA focuses on the whole life cycle of products, including production, manufacturing, use, disposal, etc. The LCA model for evaluation of CDW management could be divided into two main subsystems: collection and transportation, and treatment and disposal [22]. In the process of collecting and delivering CDW, it’s of great importance to find the most appropriate route so as to realize sustainable construction [23]. The transport distance is a significant factor to realize environmental and economic sustainability [19]. The recycling of CDW from building projects is beneficial to promoting the practice of sustainable development and activating its applications within construction sectors by emphasizing its economic and ecological benefits [24].

### 2.2. Reverse Logistics

With the enhancement of people’s awareness of sustainable development, people are committed to building an environment-friendly and resource-saving society. Due to its contribution to environmental protection and resource conservation, reverse logistics has gradually attracted the attention of academia and enterprises.

The difference between reverse logistics and traditional forward logistics lies in the flow direction of goods. In traditional forward logistics, goods flow from manufacturers to intermediaries. After the interaction of intermediaries at all levels, goods are delivered to the customers. The corresponding relationship ranges from “one to many” to “many to many”. Conversely, reverse logistics refers to the process of returning goods from the point of consumption back to the starting point of production [25]. Its corresponding relationship during the period is “many to one”. In waste management, reverse logistics refers to the process of regaining value from the generation nodes to the final disposal nodes.

Reverse logistics has attracted the attention of most researchers because of its great significance for building an environment-friendly and resource-saving society. Nowadays, waste material in reverse logistics has attracted increasing attention from both academics and practitioners. A reverse logistics network design problem was considered for end-of-life vehicles [26,27]. Bottani et al. [28] proposed an economic evaluation of several reverse logistics scenarios for collecting packaged food waste from the retail chain in Italy. A multi-objective reverse logistics network was designed to manage medical waste [29,30]. Fathollahi-Fard et al. [31] gave a sustainable framework for an integrated water supply and wastewater collection system. Reverse logistics was also applied in the sustainable management of municipal waste collection [32].

In the construction industry, the reverse logistics of CDW has become the main research object in recent years. Oliveira Neto and Correia [33] assessed the advantages of implementing reverse logistics to recycle CDW from economic and environmental perspectives. Rana and Xueqing [34] used LINGO software to solve a multi-stage network-based model for the reverse logistics management of inert construction waste. They also presented a lean thinking-based multi-layer value stream assessment approach to assess the overall reverse logistics network of inert construction waste management [35]. A hybrid Genetic Algorithm is proposed to optimize vehicle route planning for CDW collection from construction projects to recycling facilities [36]. Bi et al. [37] proposed three improvement strategies (optimal facility choice, order sequencing, and raising load ratio) to optimize CDW collection and transportation.

And for the objectives of reverse logistics, most of the research aims at profit maximization or cost minimization. For example, Erfan et al. [38] proposed a two-stage stochastic mixed-integer linear programming of reverse logistics network design considering profit maximization. Rana and Xueqing [34] considered the minimization of the total cost at starting nodes, intermediate nodes, and ending nodes which are composed of facility-based cost and non-facility-based cost. With the strengthening of environmental protection, the requirement of environmental objectives occurs. Santander et al. [39] formed a mixed-integer linear programming model for plastic recycling to realize the maximization of economic and environmental benefits, with money saved and carbon emissions reduced. Reddy et al. [40] presented a mixed-integer linear programming model to solve a green reverse logistics network incorporating carbon emission costs. Shahparvari et al. [41] developed a stochastic optimization model for reverse logistics in closed-loop supply chains to minimize the overall costs of the network, such as costs of carbon emission, as well as opening new facilities. Meanwhile, there are a few research considering social objectives. Budak [16] designed a recycling network for end-of-life mobile phones by minimizing reverse logistics costs and maximizing social goals. Safdar et al. [42] proposed a multi-objective reverse logistics network for electronic waste management which aimed to maximize profit and minimize carbon emissions as well as maximize the job opportunities in a reverse logistics network.

As mentioned above, due to the challenge of reverse logistics of CDW recycling, to meet economic, environmental, and social requirements, this paper is devoted to studying the reverse logistics of CDW recycling with the aim of minimum economic cost, minimum environmental pollution, and minimum social impact.

### 2.3. Vehicle Routing Problem

Vehicle routing problems (VRP) as one of the most critical problems in logistics network design, is a classic optimization problem in operations research, composed of multiple participants such as vehicles, depots, and customers [43]. By planning a reasonable transportation route, an objective such as minimum cost, shortest time, or shortest distance can be achieved with the requirements of customer satisfaction and other constraints (e.g., vehicle capacity limitation, vehicle quantity restriction, vehicle driving distance limitation, etc.).

In recent decades, VRP has attained massive popularity as it is close to real-life scenes. A proper solution of VRP could bring high logistics efficiency and low logistics cost. For different constraints, many subproblems have been derived from traditional VRP.

#### 2.3.1. Vehicle Routing Problem with Time Windows (VRPTW)

VRPTW is a variant of VRP considering time windows for customer satisfaction. The time window is usually divided into a hard time window and a soft time window. Under the constraint of a hard time window, if one vehicle reaches the customer early, the vehicle has to postpone the beginning of the service until the time window opens [44]. If one vehicle arrives after the end of the time window, the vehicle can only return with the undelivered goods. Besides, a soft time window is also frequently adopted. Under the constraint of a soft time window, if one vehicle arrives early or late, the vehicle can deliver goods to the customer, but certain compensation is required.

#### 2.3.2. Multi-Depot Vehicle Routing Problem (MDVRP)

MDVRP discusses the routing problem in which multiple depots provide services to customers by reasonably dispatching vehicles and selecting appropriate driving routes. Generally, exact algorithms or heuristic algorithms can be used to solve MDVRP. Exact algorithms were used to solve MDVRP studied by Contardo and Martinelli [45] and Lalla-Ruiz et al. [46]. However, heuristic techniques seem to be more viable to find the optimal solution for MDVRP [47]. There were some studies for MDVRP using heuristic algorithms, such as genetic algorithm [48,49,50], variable neighborhood search algorithm [47,51,52], and large neighborhood search algorithm [53,54]. Although the genetic algorithm has a good global search ability, it is easy to fall into local optimum, and cannot solve large-scale computational problems well. The search range of the variable neighborhood search algorithm in the solution space is large, but it also has large randomness, which is prone to producing poor results. In the large neighborhood search algorithm, the destroy operator and repair operator can be used to accelerate the generation of a better solution, but it is easy to fall into local optimal.

#### 2.3.3. Multi-Depot Vehicle Routing Problem with Time Windows (MDVRPTW)

Based on MDVRP and VRPTW, researchers began to study MDVRPTW which was firstly proposed by Cordeau et al. [55]. Dondo and Cerdá [56] proposed a hybrid local improvement algorithm for large-scale MDVRPTW to minimize total service cost. Bettinelli et al. [57] presented a branch-and-cut-and-price algorithm for the exact optimization of a multi-depot heterogeneous vehicle routing problem with a time window. Bae and Moon [58] developed a heuristic algorithm and a hybrid genetic algorithm for MDVRPTW considering delivery and installation vehicles to minimize the total relevant cost of depots, vehicles, transportation, and labor. Zhen et al. [59] studied a mixed integer programming model to minimize total traveling and service time for the last-mile distribution networks. Wang et al. [60] designed a hybrid genetic algorithm with tabu search to solve a multi-depot pickup and delivery vehicle routing problem under time window constraints. Fan et al. [61] applied a hybrid genetic algorithm with variable neighborhood search for MDVRPTW considering speed changes and road types.

In recent years, route planning for a fleet of vehicles in reverse logistics has attracted attention from researchers. Hannan et al. [62] proposed a particle swarm algorithm to determine the route optimization solutions for solid waste in a capacitated VRP model. To collect returned goods, Foroutan et al. [63] applied a simulated annealing algorithm in order to find near-optimal solutions. Emre and Umut [64] gave a short transportation route for medical waste vehicles. To recycle municipal solid waste, Mojtahedi et al. [65] built a sustainable routing optimization model based on the financial, environmental, and social goals. Marampoutis et al. [66] studied a multi-objective VRP with several real-life constraints in reverse logistics management of refillable glass bottles.

The recycling of CDW is of great importance to promote the practice of sustainable development. An optimal vehicle route planning can effectively reduce travel costs and ultimately improve waste resource efficiency. However, from the literature, little attention has been given to VRP in the construction industry, especially to the reverse logistics of CDW recycling. Hence, this paper tries to propose an optimization model on MDVRPTW for the reverse logistics process of CDW recycling from the perspectives of economic effect, environmental effect, and social effect. A method combined with a large neighborhood search algorithm and a local search algorithm is developed to plan the transportation route for CDW reverse logistics process.

## 3. Problem Description

**Figure 1:**
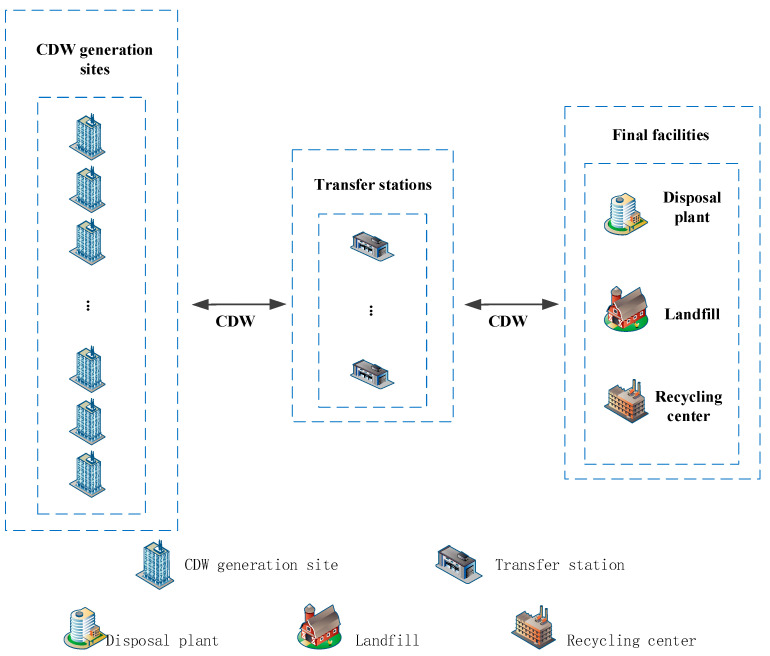
Collection system for reverse logistics of CDW.

The transfer station as an intermediate station can increase the efficiency of the waste management system [67]. The transfer station recycles and transfers CDW from scattered CDW generation sites. As shown in Figure 1, transfer stations play a vital role in the connection of CDW generation sites and final facilities. Different types of CDW are expected to be treated in specific different ways [68]. Therefore, on-site sorting is implemented at CDW generation sites, where CDW can be divided into three categories: directly recycled, reprocessed, or landfilled according to their characteristics. After that, all CDW are transported centrally to the transfer station, which can effectively save transportation costs.

**Figure 2:**
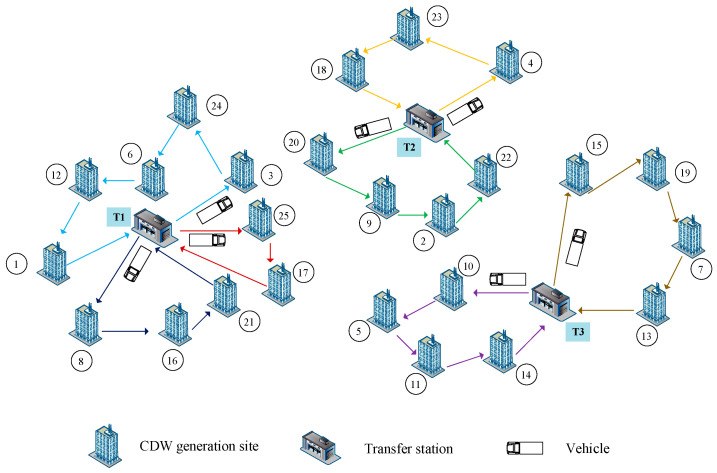
An example of MDVRPTW recycling network.

Considering an MDVRPTW of CDW recycling, a reverse logistics system is illustrated in Figure 2. The reverse logistics system of CDW in this problem is composed of two participants, including CDW generation sites and transfer stations. At CDW generation sites, CDW is generated, such as construction sites of new-build, rebuild, demolition or expansion. At transfer stations, all CDW transported from CDW generation sites are classified and collected according to CDW management specifications. An example is shown in Figure 2, there are 25 CDW generation sites, each with a random number from 1–25, and 3 transfer stations named T1, T2 and T3 respectively. CDW is transported directly from CDW generation sites to transfer stations by vehicles. A transfer station can handle CDW from multiple CDW generation sites. The relationship between them is viewed as “many-to-one”. In this paper, all vehicles start and return to transfer stations.

For this MDVRPTW, there are some assumptions given below.

(1) The location and quantity of CDW generation points, transfer stations, recycling centers, and disposal plants are known.
(2) Each vehicle departs from the transfer station at the beginning and returns to the transfer station after completing the recycling tasks.
(3) Each CDW generation site can be serviced by only one vehicle.
(4) The fleet of vehicles is homogeneous.
(5) Traffic jam is ignored.

## 4. Model and Solution Method

### 4.1. Model Construction

Due to the challenge of reverse logistics of CDW recycling that provides a CDW route plan for multiple participants and coordinates the transportation process between them, this paper is devoted to building an optimization model for MDVRPTW which could coordinate relevant CDW participants and plan a cost-effective, environment-friendly and resource-saving transportation route.

#### 4.1.1. Symbols and Notations

Hence, the proposed mathematical model takes economic, environmental, and social effects into consideration. In this paper, the economic effect is represented by time penalty cost, vehicle fixed cost, and vehicle variable cost. Besides, the environmental effect is estimated by carbon emission cost as diesel vehicles are generally used to transport CDW. The social effect is represented by the emotional compensation cost of people along the routes and sites [69]. The objective function of this proposed optimization model is to minimize the total cost consisting of economic, environmental, and social costs. The related notations are given in Table 1.

#### 4.1.2. Object Functions

As this paper discusses the economic effect, environmental effect, and social effect of MDVRPTW for the reverse logistics process of CDW recycling, the corresponding economic cost, environmental cost, and social cost are considered in this proposed model to obtain a cost-effective, environment-friendly, and resource-saving transportation plan.

Firstly, the economic costs including vehicle fixed cost, penalty cost, and vehicle variable cost are given below.

(1) Vehicle cost

Once a vehicle is put into use, the corresponding fixed costs including depreciation, repairing, and maintenance of vehicles are required. Hence, the vehicle fixed cost VC can be expressed as

(1) $$VC=\sum_{i\in G}\sum_{j\in C}\sum_{h\in H}fc_{h}v_{h}$$

(2) Penalty cost

As customers always want to be served at the right time, according to time window constraints, this paper introduces a corresponding penalty cost. $\left[e_{i},l_{i}\right]$ is set to be a desired service period for CDW generation site i, which means there is no penalty cost if the customer is served in this period. However, if the vehicle reaches earlier than $e_{i}$ or later than $l_{i}$, the corresponding penalty cost will be incurred. Moreover, if the vehicle returns to transfer stations beyond the time window, a penalty cost should also be paid. Hence, the penalty cost PC considering the time window constraint is given as

(2) $$PC=\sum_{i\in G}\max((e_{i}-t_{i}^{ha})p_{e},0)+\sum_{i\in G}\max((t_{i}^{hl}-l_{i})p_{l},0)+\sum_{j\in C}\max((t_{j}^{hb}-b_{j})p_{b},0)$$

(3) Variable cost

The variable cost of a vehicle traveling along a route mainly depends on distance and load. Transportation costs and driver wages account for a vital part of the whole transportation process.

Here, the transportation cost from node i∈G to node j∈C is as follows

(3) $$ct=\sum_{i\in G}\sum_{j\in C}q_{i}k_{ij}x_{ij}$$

The driver’s wages depend on the total traveling time of routes, which is expressed as

(4) $$dw=\sum_{i\in G}\sum_{j\in C}ddis_{ij}/v_{ij}x_{ij}$$

Hence, the total vehicle variable cost FC can be expressed as

(5) $$FC=\sum_{i\in G}\sum_{j\in C}q_{i}k_{ij}x_{ij}+\sum_{i\in G}\sum_{j\in C}ddis_{ij}/v_{ij}x_{ij}$$

Secondly, for the environmental cost, carbon emission cost is given as below.

(4) Carbon emission cost

CDW has multiple negative impacts on the environment, including land use, landfill consumption, the pollution of soil, water, and air, resource consumption, etc. In the process of recycling CDW, a large number of harmful pollutants that are harmful to the environment, such as nitrogen oxides, hydrocarbons, carbon monoxide, and particulate matter, are released. The environmental pollution caused by the driving process mainly includes two parts: solid particle pollution and gaseous gas pollution.

As total greenhouse gas emissions continue to increase, more and more serious consequences for the environment and public health are likely to occur, such as food production risks, sea-level rise hazards, and global ecological balance disruption. An increasing number of governments turn carbon neutrality into national strategies, and more than 110 countries are determined to achieve the carbon-neutral target by 2050 [70]. It is estimated by International Energy Agency that the building industry accounts for nearly 40% of total direct and indirect CO_2_ emissions. In this paper, carbon emission costs are used to estimate the bad effects of gaseous gas pollution in the transportation process for the reverse logistics of CDW recycling, which can be viewed as the tax imposed on carbon emission. As carbon emission of the transportation process comprises an empty vehicle and a loaded vehicle, based on the research of Qian and Eglese [71] and Cai et al. [72], carbon emission could be considered in direct proportion to fuel consumption. Diesel engines have found broad use in transporting CDW and a standard CO_2_ emission for diesel fuel is 2.61 kg/L [65]. Following Xiao et al. [73] and Mojtahedi et al. [65], this paper adopts fuel consumption rate (FCR) to study fuel consumption, which considers fuel consumption per unit distance under two kinds of vehicle load conditions: empty load and full load. When the vehicle’s unloaded weight is $Q_{vehicle}$ and the shipped load is $Q_{1}$, we can formulate the FCR as a linear function dependent on load $Q_{1}$ approximately as

(6) $$f\left(Q_{1}\right)=a(Q_{vehicle}+Q_{1})+b$$

Therefore, the vehicle’s FCR with a full load can be expressed as

(7) $${f^{\prime }}_{h}=aQ_{full}+b$$

The vehicle’s FCR with an empty load can be expressed as

(8) $$f_{h}=aQ_{vehicle}+b$$

By considering these two vehicle load conditions, when the shipped load is *Q*, FCR can be expressed as

(9) $$f\left(Q\right)=f_{h}+\left({f^{\prime }}_{h}-f_{h}\right)Q/cap_{h}$$

Hence, the carbon emission cost CC for a vehicle from node i to node j under a load of *Q* is formulated as

(10) $$CC=\sum_{i\in G}\sum_{j\in C}\sum_{h\in H}uw\left(f_{h}+\left({f^{\prime }}_{h}-f_{h}\right)Q/cap_{h}\right)dis_{ij}x_{ijh}$$

At last, for the social cost, the emotional compensation cost is given below.

(5) Emotional compensation cost

The use of vehicles and machinery in the recycling process may also bring some negative effects, including dust and noise pollution. The research conducted by Lampert et al. [74] explained that negative emotions including anger, anxiety, sadness, and stress could trigger atrial fibrillation. Thacher et al. [75] found that transportation noise was associated with a small increase in atrial fibrillation risk. In the process of CDW recycling, noise and dust will inevitably cause negative emotions to surround residents due to loading and unloading operations and vehicle operating. Moreover, in the transportation process, recycling vehicles will also bring some negative emotions when they come into people’s sight. Hence, emotional compensation costs could be built to realize the minimum total social effect which includes the total site effect for people living around CDW generation sites and the transportation effect for all routes between CDW generation sites and transfer stations. Based on this, the emotional compensation cost is related to population density, and can be divided into two parts: emotional compensation cost in transportation and emotional compensation cost in sites.

The emotional compensation cost in transportation is defined as

(11) $$r_{t}=\sum_{i\in G}\sum_{j\in C}\lambda p{d^{\prime }}_{ij}dis_{ij}$$

The emotional compensation cost in sites is defined as

(12) $$r_{l}=\sum_{i\in G}\lambda pd_{i}\pi \theta^{2}$$

Hence, the total negative emotional compensation cost EC can be calculated as

(13) $$EC=\sum_{i\in G}\sum_{j\in C}\lambda p{d^{\prime }}_{ij}dis_{ij}+\sum_{i\in G}\lambda pd_{i}\pi \theta^{2}$$

#### 4.1.3. The MDVRPTW Model

According to the cost analysis, the objective function with the aim to minimize the total cost TC is given as

(14) min TC=VC+PC+FC+CC+EC

There is

(15) $$\begin{array}{l} =\overset{\min}{\underset{i\in G}{\sum }}\overset{TC}{\underset{j\in C}{\sum }}\underset{h\in H}{\sum }fc_{h}v_{h}+\underset{i\in G}{\sum }\max((e_{i}-t_{i}^{ha})p_{e},0)+\underset{i\in G}{\sum }\max((t_{i}^{hl}-l_{i})p_{l},0)\\ +\underset{j\in C}{\sum }\max((t_{j}^{hb}-b_{j})p_{b},0)+\underset{i\in G}{\sum }\underset{j\in C}{\sum }q_{i}k_{ij}x_{ij}+\underset{i\in G}{\sum }\underset{j\in C}{\sum }ddis_{ij}/v_{ij}x_{ij}\\ +\underset{i\in G}{\sum }\underset{j\in C}{\sum }\underset{h\in H}{\sum }uw\left(f_{h}+\left({f^{\prime }}_{h}-f_{h}\right)Q/cap_{h}\right)dis_{ij}x_{ijh}+\underset{i\in G}{\sum }\underset{j\in C}{\sum }\lambda p{d^{\prime }}_{ij}dis_{ij}\\ +\underset{i\in G}{\sum }\lambda pd_{i}\pi \theta^{2} \end{array}$$

Subject to

(16) $$\sum_{i\in G}\sum_{j\in C}x_{ijh}=1,\forall h\in H$$

(17) $$\sum_{i\in C}\sum_{j\in C}x_{ijh}=1,\forall h\in H$$

(18) $$\sum_{i\in G}\sum_{j\in C}x_{ijh}-\sum_{i\in G}\sum_{j\in C}x_{jih}=0,\forall h\in H$$

(19) $$\sum_{i\in G}\sum_{j\in C}q_{i}x_{xjh}\leq cap_{h},\forall h\in H$$

(20) $$e_{i}\leq t_{i}^{ha}\leq l_{i},\forall i\in G,\forall h\in H$$

(21) $$t_{j}^{hb}\leq b_{j},\forall j\in C,\forall h\in H$$

(22) $$x_{ijh}\in \left\{0,1\right\},\forall i\in G,\forall j\in C,\forall h\in H$$

(23) $$y_{jh}\in \left\{0,1\right\},\forall j\in C,\forall h\in H$$

Here, Equation (16) ensures that all CDW generation sites are visited once. Equation (17) guarantees no vehicle can travel between two transfer stations. Equation (18) means each vehicle begins and finishes at the same node. Equation (19) guarantees a load of vehicles is no more than its capacity. Equation (20) is the time windows constraint of CDW generation site i (i∈G). Equation (21) is the time constraint of transfer station j (j∈C). Equations (22) and (23) state the binary restrictions of the decision variables.

### 4.2. Model Solution

VRP and its variants derived from the Traveling Salesman Problem (TSP) belong to NP-hard problems [76]. Adaptive large neighborhood search (ALNS) is a variant of large neighborhood search, which was proposed by Ropke and Pisinger [77] firstly. Yu et al. [78] proposed that ALNS had an excellent performance in solving various real-world transportation problems, such as the orienteering problem and vehicle routing problems. According to the literature review, the ALNS is one of the best-preferred algorithms to address the MDVRPTW. Hence, this paper introduces a hybrid adaptive neighborhood search algorithm (HALNS) to solve the VRP from multiple CDW generation sites to multiple transfer stations.

This paper proposes two destroy operators (i.e., random destroy and worst destroy) and three repair operators (i.e., random repair, greedy repair, and regret repair). Besides the multiple groups of destroy operators and repair operators, seven types of local search operators are used to expand the search range of solution space. An adaptive method is applied to update the weight of the operators, and the acceptance criteria of the simulated annealing framework are used to further optimize the initial solution until the maximum number of iterations is reached.

**Figure 3:**
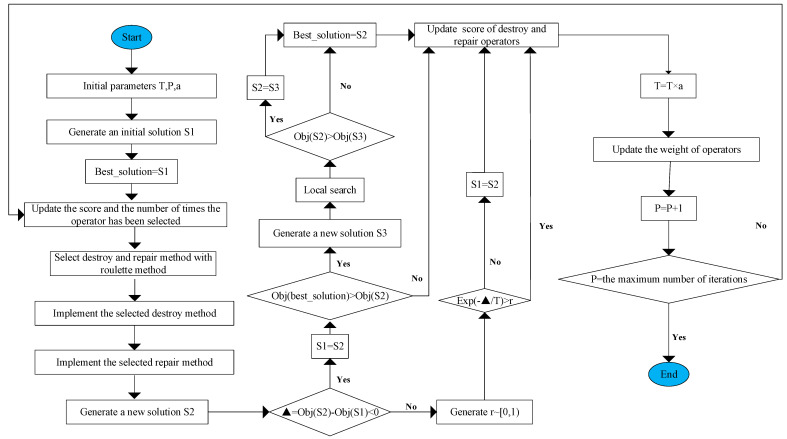
The flowchart of HALNS.

Figure 3 shows the flow chart of HALNS. In the beginning, the initial parameters are set and the initial solution S1 is generated which is taken to be the optimal solution. Then, it comes to the iterative process. In each iteration, scores of the destroy operator, the repair operator, and the selected times are updated. The roulette method is used to select destroy and repair operators. After performing the destroy and repair operations, a new solution S2 is generated. By comparing S2 and S1 as well as the value of the objective function, scores of the destroy operator and repair operator are dynamically adjusted. This process is accompanied by the updated optimal solution, and the idea of simulated annealing is used to accept the poor solution with probability, where T represents the initial temperature, and α represents the cooling ratio. When the number of iterations and the maximum number of iterations are equal, the algorithm stops.

#### 4.2.1. Initiation

Firstly, the maximum number of iterations P, the initial temperature T, and the cooling rate α are set. In the initial stage of annealing, a large value T is used to make all transition states acceptable at the beginning. T is gradually reduced with α as the annealing progresses. α is a positive number less than 1, generally between 0.8 and 0.99. Then the generated point sequence is randomly shuffled. By considering the constraints of vehicle capacity and time window, vehicles are allocated to transfer stations. Finally, the initial vehicle driving route and the required number of vehicles can be obtained.

#### 4.2.2. Destroy Operators

**Figure 4:**
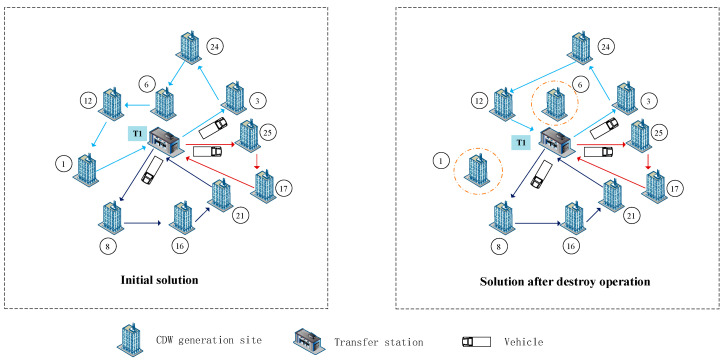
The process of the destroy operation.

Two destroy operators: random destroy and worst destroy are performed within the proposed HALNS algorithm, which is explained as follows. An initial feasible solution becomes an incomplete solution after performing destroy operation. It could be seen in Figure 4, that a shorter route with two CDW generation sites unserved by vehicles is obtained after performing destroy operation. The initial solution in Figure 4 is a part of Figure 2, with 10 CDW generation sites and a transfer station T1.

(1) Random destroy

The random destroy aims to increase the diversity of the search process. In this paper, a random number between $r_{min}$ and $r_{max}$ is generated, where $r_{min}$ represents the minimum degree of destruction and $r_{max}$ represents the maximum degree of destruction. Then, this random number is multiplied by the number of CDW generation points, and the number of generation points to be removed by rounding is obtained. Finally, CDW generation points of these quantities are removed randomly.

(2) Worst destroy

The purpose of worst destroy is to remove the CDW generation points with great contribution to the objective. First, calculate the difference between the original objective value and the objective value after removing a CDW generation point. Then randomly generate a number between $r_{min}$ and $r_{max}$, and calculate the product of this number and the number of CDW generation points. Finally, sort the differences in descending order, and remove the previous part of the CDW generation points of the product number.

#### 4.2.3. Repair Operators

**Figure 5:**
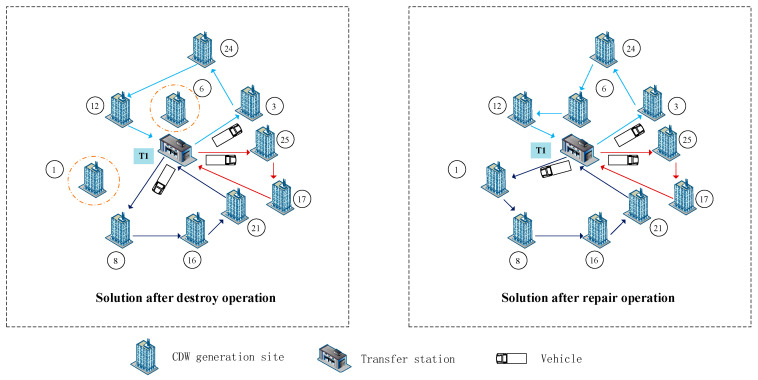
The process of the repair operation.

The repair operation can make the solution after the destroy operation to be feasible. Three repair operators: random repair, greedy repair, and regret repair are presented below. After the destroy operation in Figure 4, Figure 5 shows a better solution through the repair operation which shows the solution after the reinsertion of the previously unserved CDW generation sites into tours.

(1) Random repair

The removed CDW generation points are inserted into the assigned node sequence randomly.

(2) Greedy repair

The removed CDW generation points are inserted into the assigned node sequence in turn. The objective function is calculated to obtain the increment size of each solution. Then, the CDW generation point with the smallest increment and the insertion position is selected until all the removed CDW generation points are reinserted.

(3) Regret repair

The difference in the objective function value between the optimal solution and the solution when the CDW generation point is inserted back to the σ sub-optimal position is calculated. After summing the difference up, the CDW generating point with the largest sum and its optimum location is selected.

#### 4.2.4. Roulette Selection

The roulette wheel method is used to select destroy operator and repair operator. The probability that the operator is selected is proportional to its weight. First, the weight $w_{i}$ of each operator is calculated. Hence, the proportion $p_{i}$ of each operator in the total weight of all operators can be obtained as

(24) $$p_{i}=w_{i}/\sum_{i=1}^{n}w_{i}$$

Then, the cumulative weight $q_{j}$ of each operator is calculated as

(25) $$q_{j}=\sum_{j=1}^{i}p_{j}$$

Finally, a sample value r∈[0,1) is generated randomly. If $q_{i}>r$, the i-th operator is selected.

#### 4.2.5. Local Search

**Figure 6:**
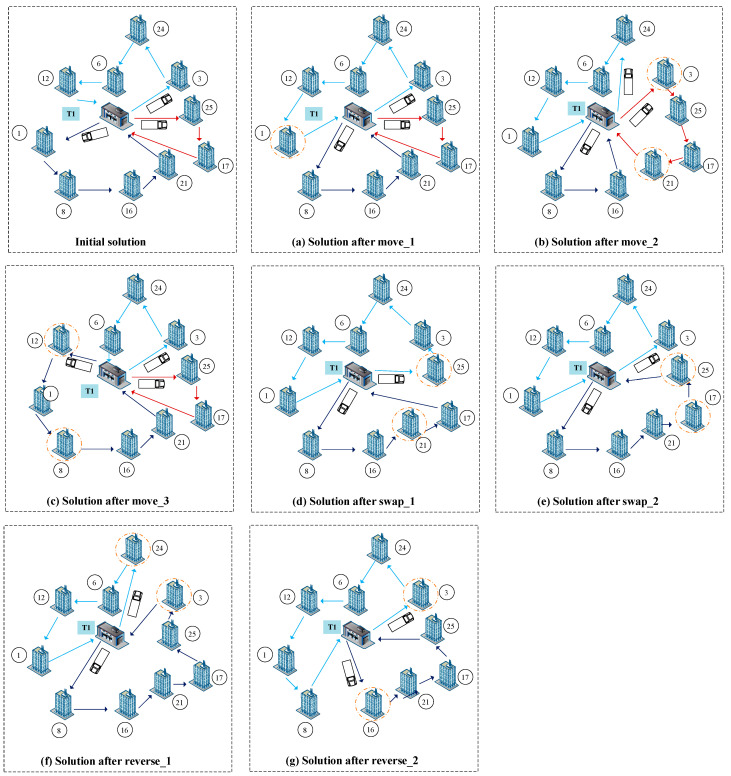
New solution after local search operators.

In order to get better solutions, seven types of local search operators are proposed. Figure 6 represents an example, and some local search operators are implemented on the initial solution. The initial solution in Figure 6 is a part of Figure 2, with 10 CDW generation sites and a transfer station T1.

**Move_1**: select a node in the current solution randomly and remove this node to a new position after the node randomly. An example is given in Figure 6a, node 1 is selected randomly, and a new solution is obtained.

**Move_2:** select two non-adjacent nodes in the current solution randomly and move the nodes between the selected first node and the second node backward in one position. Then, move the selected second node to the position after the selected first node. As shown in Figure 6b, node 3 and node 21 are randomly selected to obtain a new solution.

**Move_3**: select two non-adjacent nodes in the current solution randomly and move the nodes between the selected first node and the selected second node forward. Then, move the selected first node to the position after the selected second node. As shown in Figure 6c, node 12 and node 8 are selected randomly, and a new solution is obtained.

**Swap_1**: select two nodes in the current solution randomly and exchange the positions of the two nodes. Node 21 and node 25 are selected randomly, and a new solution is obtained in Figure 6d. The number of vehicles is reduced from three to two.

**Swap_2**: select two nodes in the current solution randomly and swap the selected second node with its following nodes. Then, swap the selected first node with its following nodes. In Figure 6e, node 25 and node 17 are selected randomly to get a new solution.

**Reverse_1**: select two nodes in the current solution randomly and exchange the positions of these two nodes and reverse the nodes between the swapped ones. Node 3 and node 24 are selected randomly in Figure 6f, and a new solution is obtained.

**Reverse_2**: in addition to Reverse_1, reverse the selected second node and its following nodes first, then reverse the selected first point and its following nodes. The final solution is shown in Figure 6g, the node 3 and node 16 are selected randomly.

#### 4.2.6. Adaptive Mechanism

At first, all operators have the same weight and score. In the iterative process, the score is given in a stepwise manner according to the different performances of the operator, and the score is proportional to the performance of the operators. Then, this paper sets four classifications as follows:

The new solution S2 is better than the current solution S1 and the best solution Sb, that is, the solution is updated by S1 = S2 and Sb = S2 after destroying or repairing the operator. In this case, $r_{1}$ is added to the score of operators.

The new solution S2 is worse than the current best solution, Sb, but better than the current solution S1, that is, the current solution is updated by S1 = S2 after destroying or repairing the operator. In this case, $r_{2}$ is added to the score of operators.

Although the new solution S2 is worse than the current solution S1, it satisfies the Metropolis criterion of simulated annealing algorithm, that is, the current solution S1 is updated by S1 = S2. In this case, $r_{3}\;$ is added to the score of operators.

The new solution S2 is worse than the current solution S1, and it does not meet the Metropolis criteria of simulated annealing algorithm, that is, the new solution S2 is not accepted. In this case, $r_{4}$ is added to the score of operators.

Finally, the weight of operators is updated adaptively so as to improve the optimization ability of this proposed algorithm, shown as

(26) $$w_{i}=\{\begin{array}{cc} w_{i} & ,s_{i}=0\\ \left(1-\beta \right)w_{i}+\beta \left(s_{i}/u_{i}\right) & ,s_{i}>0 \end{array}$$

where $s_{i}$ is the operator score and $u_{i}$ is the number of times the operator is used. β is the reaction factor of action weight which influences the speed of weight change, where β∈[0,1]. The larger the value of β is, the more the weight depends on the previous performance of the operator.

#### 4.2.7. Metropolis Criteria

After performing destroy and repair operations, a new solution S2 is generated. Comparing the value of the objective function solved by S2 and the original solution S1, there is θ=obj(S2)−obj(S1), where θ is the difference. The probability of accepting the new solution S2 is determined according to the size of θ and 0.

(27) $$p=\{\begin{array}{cc} 1 & ,\theta <0\\ Exp\left(-\theta /T\right) & ,\theta \;\geq 0 \end{array}$$

If θ<0, the new solution is accepted as the current solution (i.e., S1 = S2). Otherwise, the new solution is accepted under probability Exp(−θ/T). Using the Metropolis criteria to accept inferior solutions at a certain probability can avoid falling into the local optimum.

## 5. Numerical Experiments

### 5.1. Parameter Setting

After a series of testing experiments, the parameters of MDVRPTW are set, as shown in Table 2.

The difference between HALNS and ALNS lies in that ALNS does not implement local search operators. Therefore, the parameters for ALNS and HALNS are the same as shown in Table 3.

Compared with a genetic algorithm, an adaptive genetic algorithm (AGA) can adaptively adjust the crossover probability and mutation probability to maintain the population diversity and ensure the convergence of the algorithm. Hence, the fitness function in this paper is described as

(28) $$f_{i}=\frac{1}{TC_{i}}$$

The parameters of AGA are given: the number of the initial population pop is 100, the number of iterations n is 5000, the selection probability s is 0.9, the minimum crossover probability $pc_{1}$ is 0.1, the maximum crossover probability $pc_{2}$ is 0.9, the minimum mutation probability $pm_{1}$ is 0.001, the maximum mutation probability $pm_{2}$ is 0.1.

Then, the crossover probability can be calculated as

(29) $$pc=\{\begin{array}{cc} \frac{pc_{1}+pc_{2}}{2}+\frac{pc_{1}-pc_{2}}{2}\sin(\frac{f-f_{avg}}{f_{max}-f_{avg}}\cdot \frac{\pi }{2}) & ,f_{avg}\leq f\\ pc_{1} & ,f_{avg}>f \end{array}$$

And the mutation probability can be calculated as

(30) $$pm=\{\begin{array}{cc} \frac{pm_{1}+pm_{2}}{2}+\frac{pm_{1}-pm_{2}}{2}\sin(\frac{f-f_{avg}}{f_{max}-f_{avg}}\cdot \frac{\pi }{2}) & ,f_{avg}\leq f\\ pm_{1} & ,f_{avg}>f \end{array}$$

where $f_{avg}$ is the average fitness, and $f_{max}$ is the maximum fitness.

### 5.2. Basic Data

In this paper, the dataset proposed by Cordeau et al. [79] is applied. The dataset is composed of 20 instances (pr01–pr20), which originated from 10 instances (p01–p10) established by Cordeau et al. [80]. The time windows for MDVRPTW are set randomly. Set1 (pr01–pr10) adopts tighter time windows, and Set2 (pr11–pr20) adopts wider time windows.

Table 4 shows the data of pr01. There are four transfer stations and 48 CDW generation sites. The first column shows the name of nodes. The other columns give their location coordinates (X, Y), amount of CDW (Q), the earliest time to start service (ET), the last time to start service (LT), and service time (T).

### 5.3. Results Discussion

In this section, we analyze the performance of proposed HALNS with ALNS and AGA first. After that, we examine the effects of different time windows on transport routes using two sets. Based on benchmark pr01, the sensitivity analysis is conducted to explore the impact of vehicle capacity and carbon tax rate.

#### 5.3.1. Performance Analysis

The computational results tested on dataset pr01 of AGA, ALNS, and HALNS are shown in Table 5. It can be seen that HALNS performs better than AGA and ALNS. The total cost of HALNS is minimum in the optimal results and is 22.20% and 3.83% less than AGA and ALNS respectively. Under the optimal results, the vehicle cost, the time penalty cost, the variable cost, the carbon emission cost, and the emotional compensation cost of HALNS are all less than AGA and ALNS. In the average results, the total cost of HALNS improves by −23.43% and −1.08% than AGA and ALNS. About four vehicles are needed for ALNS and HALNS under the average results but six vehicles for AGA. The vehicle cost, the time penalty cost, the variable cost, the carbon emission cost, and the emotional compensation cost of HALNS are 33.33%, 63.51%, 20.35%, 20.35%, and 6.83% less than AGA respectively. The vehicle cost, the time penalty cost, the variable cost, the carbon emission cost, and the emotional compensation cost of ALNS are 0.81%, 20.99%, 1.01%, 1.01%, −0.95% greater than HALNS respectively. The reason why the emotional compensation cost of ALNS is lower than HALNS may be its high population density.

**Figure 7:**
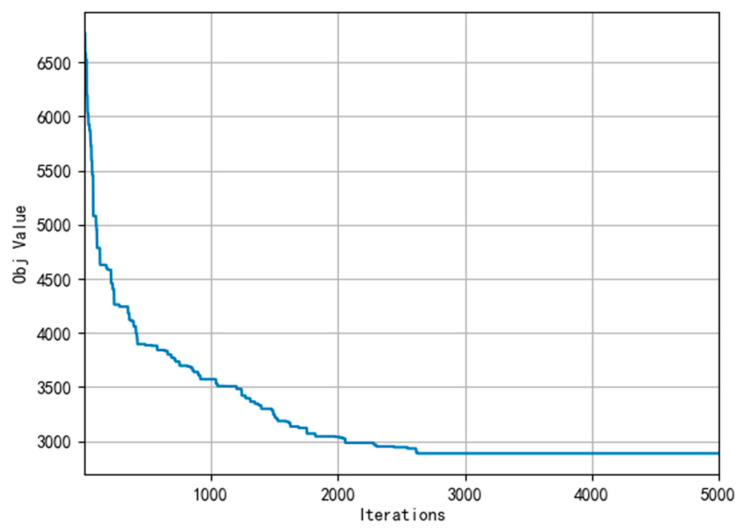
Convergence curve of AGA.

**Figure 8:**
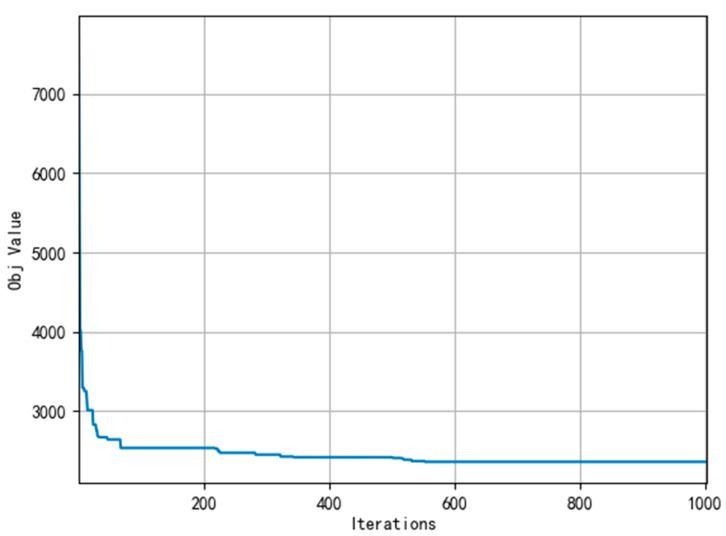
Convergence curve of ALNS.

**Figure 9:**
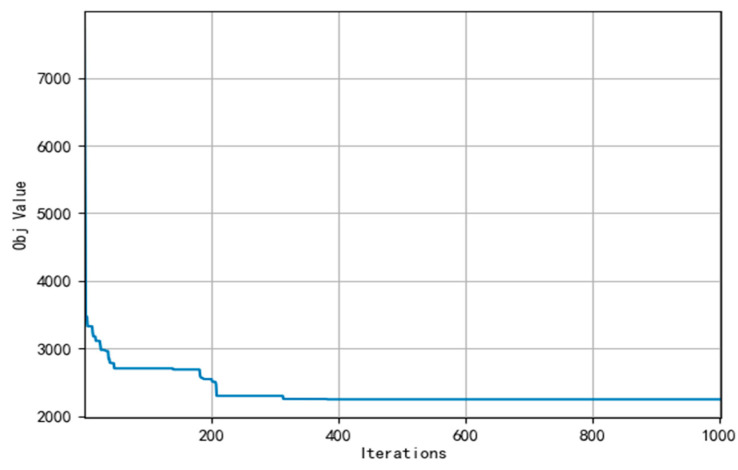
Convergence curve of HALNS.

**Figure 10:**
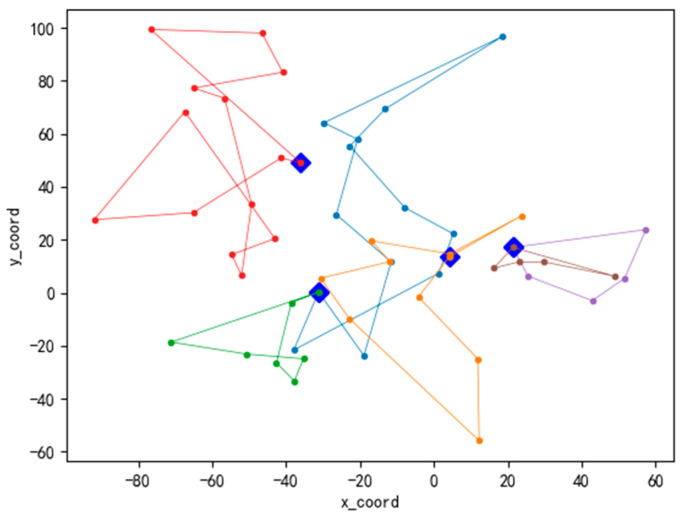
Optimal route of pr01 benchmark.

Meanwhile, Figure 7, Figure 8 and Figure 9 present the convergence curve respectively which state HALNS has better convergence than AGA and ALNS. Under the AGA algorithm, convergence occurs at about 2700 iterations, while 600 iterations for ALNS, and 400 iterations for HALNS, which further verifies the optimization ability of the ALNS algorithm. The result of the optimal route obtained by HALNS is given in Figure 10. As we can see, in the case of 48 CDW generation sites and four transfer stations, six routes are required to reach the optimal solution. The six routes are as follows:

Route 1: T1-9-42-46-39-2-44-31-37-22-T1.

Route 2: T2-27-3-48-11-T2.

Route 3: T2-34-10-45-6-T2.

Route 4: T3-13-33-20-8-29-26-15-25-23-5-17-18-16-T3.

Route 5: T4-30-12-21-24-38-40-T4.

Route 6: T5-47-35-7-41-28-4-19-1-14-36-32-43-T5.

#### 5.3.2. Time Window Analysis

As the choice of time window greatly affects the solution of the transportation plan, the experiments are made with the datasets: Set1 (pr01, pr02, pr07, pr08) and Set2 (pr11, pr12, pr17, and pr18). The results are shown in Table 6 presents different results of eight datasets under three algorithms. The results illustrate the effectiveness of HALNS is superior to AGA and ALNS in each dataset. Compared with Set2, the vehicle cost, the time penalty cost, the variable cost, the carbon emission cost, the emotional compensation cost, and the total cost of Set1 are higher than Set2 due to its tight time windows.

**Figure 11:**
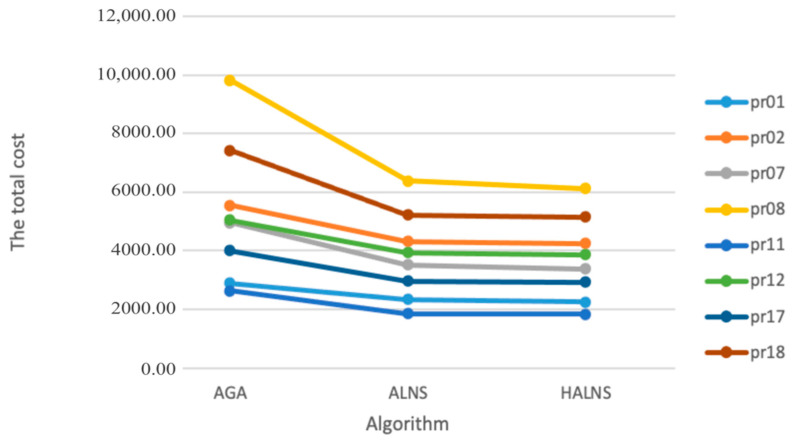
Comparison of experimental results with different datasets.

The comparison result with different datasets is illustrated in Figure 11. The total cost of these datasets can be ranked from lowest to highest as follows: pr11, pr01, pr17, pr07, pr12, pr02, pr18, pr08. Of these datasets, the dataset pr08 has the highest cost because it has the highest number of CDW generation sites and transfer stations as well as a tighter service time window. The cost of dataset pr11 is the lowest since it has the least number of CDW generating sites and transfer stations and a looser service time window. It can be seen that, for datasets with the same generation point and transfer station, the total cost curve of Set 2 is lower than that of Set1 which illustrates that a wider time window has a better economy.

#### 5.3.3. Vehicle Capacity Analysis

**Figure 12:**
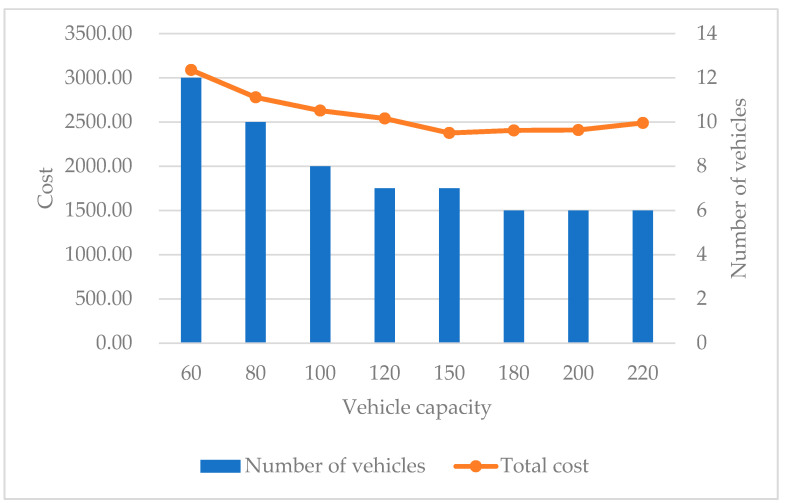
The total cost and number of vehicles with different vehicle capacities.

In real situations, different companies may use different types of vehicles to complete transportation tasks, hence vehicle capacity is viewed as an important factor in the transportation plan. In this section, a sensitivity analysis is conducted to explain the impacts of different vehicle capacities. Figure 12 shows the relationship between total cost, number of used vehicles, and vehicle capacity when the vehicle capacity ranges from 60 to 220. It can be seen that vehicle capacity affects the number of vehicles used, which in turn affects the total cost. The total cost decreases gradually as the vehicle capacity increases at the beginning. When the vehicle capacity reaches 150, the cost curve started to show an upward trend. Within a certain range, the number of vehicles used is inversely proportional to the vehicle capacity, until the saved vehicle fixed cost cannot make up for the increased transportation cost and time penalty cost. If the vehicle capacity is too small, it will increase the number of vehicles used and increase the fixed vehicle cost. However, once the vehicle capacity becomes too large, it would cause a waste of resources. Thus, the use of vehicles with reasonable capacity is beneficial to saving costs and avoiding waste.

#### 5.3.4. Carbon Tax Rate Analysis

**Figure 13:**
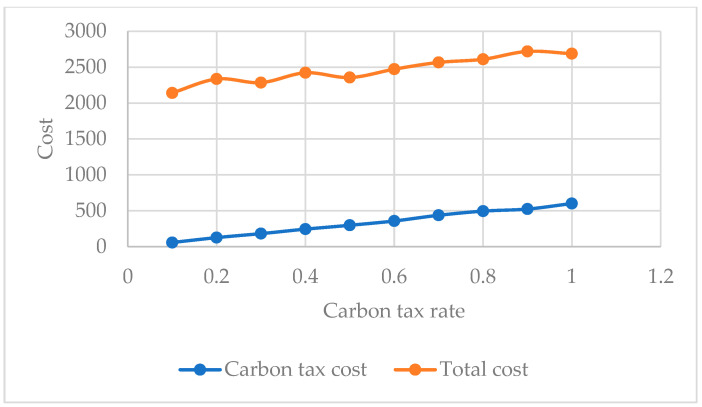
The carbon emission cost and total cost with different carbon tax rates.

Since different countries are at different stages of development, carbon tax rates vary greatly. The implementation of the carbon tax on carbon emissions plays a positive role in environmental protection. In this section, a sensitivity analysis is conducted to explain the impacts of different carbon tax rates. Figure 13 shows the relationship between total cost, carbon emission cost, and carbon tax rate when the carbon tax rate ranges from 0.1 to 1. It can be seen that when the carbon tax rate ranges from 0.1 to 1, the total cost and carbon emission cost have been on an upward trend.

**Figure 14:**
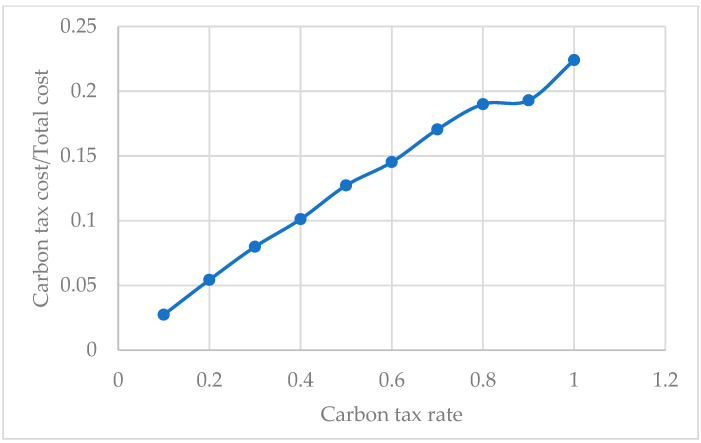
The ratio of carbon emission cost/total cost with different carbon tax rates.

Figure 14 illustrates the relationship between the ratio of carbon emission cost/total cost and carbon tax rate. It is obvious that the larger the carbon tax rate is, the larger the ratio of carbon emission cost/total cost is. This indicates that the increased carbon tax rate will lead to a change in other costs. When the carbon tax rate is relatively small, companies may sacrifice carbon emissions to gain higher profits in other areas. Otherwise, when the carbon tax rate is high, although carbon emissions are reduced, the burden on the company increases. Therefore, a reasonable carbon tax rate should be determined to achieve a cost-effective, environment-friendly, and resource-saving transportation plan.

## 6. Managerial Implications

It is of great importance for the government and enterprises to pay more attention to the recycling of CDW. The model proposed in this paper can support the decision-making of resource utilization maximization for enterprises and carbon emission management for the government.

Nowadays, it is feasible to predict the output of CDW in advance by means of information technology. Therefore, the enterprise can determine the fleet size and formulate the optimal vehicle route in advance according to the geographical location and output of the CDW generation sites. The fixed cost and fuel consumption cost of vehicle use can be effectively reduced by reasonably allocating vehicles of different capacities to close facilities as it maximizes vehicle utilization.

The objective function of the model in this paper takes into account the cost of carbon emissions in the transportation process, which can provide strong support for government organizations to make decisions. However, raising the price per unit of emissions does not always lead to environmental improvements. In order to better achieve carbon peaking and carbon neutrality, local government organizations should set a reasonable price for carbon tax according to the actual situation of each region.

## 7. Conclusions

The recycling of CDW is considered a vital stage to mitigate CDW impacts due to the huge negative impacts on the economy, environment, and society. This paper studies transportation routes among multiple CDW generation sites and transfer stations in the recycling process of CDW. We propose an optimization model for MDVRPTW of CDW recycling to coordinate relevant participants and suggest a cost-effective, environment-friendly, and resource-saving transportation plan. Under the constraints of the time window and vehicle capacity, the distribution and transportation route between CDW generation sites and transfer stations are studied, while the transportation process for reverse logistics of CDW recycling is discussed from the perspectives of economic effect, environmental effect, and social effect. From the numerical experiments, we can reach the following conclusions: (1) The tighter the service time window, the higher the total cost. It’s feasible to take advantage of information technology to plan transportation time in advance and improve service levels. (2) The capacity of recycling vehicles has an impact on the transportation plan. When it doesn’t meet its optimal capacity, the total cost will not be the lowest. The allocated capacity and vehicle routes are conductive to achieve the maximum utilization of vehicles. (3) The carbon tax rate within a certain range has a positive effect on reducing carbon emissions.

From the perspective of sustainability, HALNS is proposed to solve the optimization model, and the computational results show its better performance. The total cost of HALNS is minimum in the optimal results and is 22.20% and 3.83% less than AGA and ALNS respectively. In the average results, the total cost of HALNS improves by −23.43% and −1.08% than AGA and ALNS. Through the sensitivity analysis of vehicle capacity and carbon tax rate, the results can support the decision-making for resource-saving, waste reduction, and environmental protection. It is feasible for enterprises to reduce economic, environmental, and social impacts by controlling the capacity of recycling vehicles. The government should introduce a reasonable carbon tax policy to achieve a cost-effective, environment-friendly, and resource-saving transportation plan as a higher price per unit of emissions does not always lead to greater efficiency.

In future work, heterogeneous vehicles would be considered for reverse logistics of the CDW recycling process. Other algorithms are also considered to be hybridized with ALNS to solve MDVRPTW to obtain better results. Another extension is to take the facility location of transfer stations and transportation routing into consideration simultaneously.

## Figures and Tables

**Table 1:** Related notations for pr01 benchmark instance.

**Sets:**
N=(V, A) the transportation network of nodes *V* and arcs *A*
G={1,2…,g} the CDW generation sites, G∈V
C={1,2,…,c} the transfer stations, C∈V
H={1,2,…,h} the fleet of collection vehicles
**Parameters:**
$f_{h}$ : the fuel consumption per unit distance of an empty vehicle h∈H
${f^{\prime }}_{h}$: the fuel consumption per unit distance of a vehicle h∈H with a full load
k: the unit fuel consumption cost
$fc_{h}$ : the fixed cost for using a vehicle h∈H
d: the driver cost per time unit
$cap_{h}$ : the maximum capacity of the vehicle h∈H
$dis_{ij}$ : the traveling distance from the node i∈G to node j∈C
$v_{ij}$ : the traveling speed between node i∈G and node j∈C
u: the carbon tax rate
w: the carbon emissions per unit of fuel consumed
$pd_{i}$ : the population density in the neighborhood of the node i∈G
$\theta_{i}$ : the impact radius of the node i∈G
$p{d^{\prime }}_{ij}$ : the population density in the link from a node i∈G to node j∈C
λ: the emotional compensation cost of each person
$q_{i}$ : the amount of CDW at the CDW generation site i∈G
$e_{i}$ : the earliest service time of CDW generation site i∈G
$l_{i}$ : the latest service time of the CDW generation site i∈G
$b_{j}$ : the latest service time of the transfer station j∈C
$t_{i}$ : the service time pe of CDW generation site i∈G
pe $:\;\mathrm{the}\;\mathrm{penalty}\;\mathrm{cost}\;\mathrm{per}\;\mathrm{unit}\;\mathrm{time}\;\mathrm{for}\;\mathrm{vehicles}\;\mathrm{arriving}\;\mathrm{earlier}\;\mathrm{than}\;e_{i}$ at the CDW generation site i∈G
pl $:\;\mathrm{the}\;\mathrm{penalty}\;\mathrm{cost}\;\mathrm{per}\;\mathrm{unit}\;\mathrm{time}\;\mathrm{for}\;\mathrm{vehicles}\;\mathrm{arriving}\;\mathrm{later}\;\mathrm{than}\;l_{i}$ at the CDW generation site i∈G
pb $:\;\mathrm{the}\;\mathrm{penalty}\;\mathrm{cost}\;\mathrm{per}\;\mathrm{unit}\;\mathrm{time}\;\mathrm{for}\;\mathrm{vehicles}\;\mathrm{arriving}\;\mathrm{later}\;\mathrm{than}\;b_{j}$ at the transfer station j∈C
$t_{i}^{ha}$: the time when the vehicle *h* reaches the CDW generation site i∈G
$t_{i}^{hl}$: the time when the vehicle *h* leaves the CDW generation site i∈G
$t_{j}^{hb}$: the time when the vehicle *h* goes back to the transfer station j∈C
**Decision variables:**
$x_{ijh}$: equals to 1 if the vehicle *h* travels from node *i* to *j*; 0 otherwise
$v_{h}$: equals to 1 if the vehicle *h* is used; 0 otherwise

**Table 2:** The relevant parameters of the model.

Parameters	Value
$f_{h}$	0.16 L/Km
${f^{\prime }}_{h}$	0.2 L/Km
k	5.6 RMB/L
$fc_{h}$	80 RMB
w	2.61 kg/L
u	0.5 RMB/kg
pe	1 RMB/min
pl	1 RMB/min
pb	2/3 RMB/min
λ	1.3 RMB/person

**Table 3:** The relevant parameters of HALNS.

Parameters	Description	Value
P	The maximum number of iterations	1000
T	The initial temperature	1000
α	The cooling ratio	0.99
β	The reaction factor of action weight	0.1
$r_{min}$	The minimum degree of destruction	0.1
$r_{max}$	The maximum degree of destruction	0.4
σ	The number of best insertions	5
$r_{1}$	The operator score when *θ* < 0 and Sb ≥ S2	15
$r_{2}$	The operator score when *θ* < 0 and Sb < S2	9
$r_{3}$	The operator score when *θ* ≥ 0, but accept	4
$r_{4}$	The operator score when *θ* ≥ 0, but does not accept	1

**Table 4:** Information for pr01 benchmark instance.

Node	X	Y	Q	ET	LT	T
T1	4.163	13.559		0	1000	
T2	21.387	17.105		0	1000	
T3	−36.118	49.097		0	1000	
T4	−31.201	0.235		0	1000	
1	−29.73	64.136	12	399	525	2
2	−30.664	5.463	8	121	299	7
3	51.642	5.469	16	389	483	21
4	−13.171	69.336	5	204	304	24
5	−67.413	68.323	12	317	458	1
6	48.907	6.274	5	160	257	17
7	5.243	22.26	13	170	287	6
8	−65.002	77.234	20	215	321	5
9	−4.175	−1.569	13	80	233	7
10	23.029	11.639	18	90	206	1
11	25.482	6.287	7	397	525	4
12	−42.615	−26.392	6	271	420	10
13	−76.672	99.341	9	108	266	2
14	−20.673	57.892	9	340	462	16
15	−52.039	6.567	4	226	377	23
16	−41.376	50.824	25	446	604	18
17	−91.943	27.588	5	444	566	3
18	−65.118	30.212	17	434	557	15
19	18.597	96.716	3	319	460	13
20	−40.942	83.209	16	192	312	10
21	−37.756	−33.325	25	414	572	4
22	23.767	29.083	21	371	462	23
23	−43.03	20.453	14	378	472	20
24	−35.297	−24.896	19	308	477	10
25	−54.755	14.368	14	329	444	4
26	−49.329	33.374	6	269	377	2
27	57.404	23.822	16	398	494	23
28	−22.754	55.408	9	257	416	6
29	−56.622	73.34	20	198	294	8
30	−38.562	−3.705	13	375	467	10
31	−16.779	19.537	10	200	338	7
32	−11.56	11.615	16	456	632	1
33	−46.545	97.974	19	72	179	21
34	16.229	9.32	22	182	282	6
35	1.294	7.349	14	159	306	4
36	−26.404	29.529	10	321	500	13
37	4.352	14.685	11	322	430	9
38	−50.665	−23.126	15	443	564	22
39	−22.833	−9.814	13	207	348	22
40	−71.1	−18.616	15	457	588	18
41	−7.849	32.074	8	203	382	10
42	11.877	−24.933	22	75	167	25
43	−18.927	−23.73	24	459	598	23
44	−11.92	11.755	3	174	332	4
45	29.84	11.633	25	130	225	9
46	12.268	−55.811	19	169	283	17
47	−37.933	−21.613	21	115	232	10
48	42.883	−2.966	10	414	531	17

**Table 5:** Comparison results of three algorithms.

	Algorithm	VC	PC	FC	CC	SC	TC
Optimal	AGA	720	47	1468.41	342.19	310.75	2888.34
	ALNS	480	77	1242.24	289.49	247.81	2336.54
	HALNS	**480**	**40**	**1207.49**	**281.39**	**238.11**	**2246.99**
Average	AGA	746.67	88	1585.79	369.55	295.11	3085.12
	ALNS	501.82	40.64	1275.99	297.35	272.34	2388.13
	HALNS	**497.78**	**32.11**	**1263.08**	**294.34**	**274.94**	**2362.26**

Note: Optimal is the best solution obtained of 10 runs, Average is the average result of 10 runs of the algorithm. The best result of the three algorithms is bold.

**Table 6:** Comparison results of different benchmarks.

Dataset	Algorithm	VC	PC	FC	CC	SC	TC
pr01	AGA	720	47	1468.41	342.19	310.75	2888.34
*m* = 48, *n* = 4	ALNS	480	77	1242.24	289.49	247.81	2336.54
	HALNS	480	40	1207.49	281.39	238.11	2246.99
pr02	AGA	960	173	2683.94	741.97	990.06	5548.97
*m* = 96, *n* = 4	ALNS	800	23	2159.89	503.33	824.36	4310.59
	HALNS	720	24	2197.14	512.01	798.70	4251.86
pr07	AGA	1520	165	2113.70	492.57	661.60	4952.87
*m* = 72, *n* = 6	ALNS	720	24	1792.87	417.80	568.21	3522.88
	HALNS	640	47	1746.81	407.07	537.94	3378.82
pr08	AGA	2240	250	4811.90	1121.34	1399.59	9822.83
*m* = 144, *n* = 6	ALNS	1360	51	3190.27	743.45	1044.25	6388.96
	HALNS	1280	65	3107.79	724.23	953.09	6130.10
pr11	AGA	640	34	1367.90	318.77	273.64	2634.32
*m* = 48, *n* = 4	ALNS	400	5	1006.22	234.49	203.90	1849.61
	HALNS	400	11	978.24	227.96	208.70	1825.91
pr12	AGA	1200	68	2469.82	575.56	728.64	5042.02
*m* = 96, *n* = 4	ALNS	880	73	1917.86	446.93	618.91	3936.71
	HALNS	880	5	1921.47	447.77	611.49	3865.74
pr17	AGA	880	3	2044.53	476.45	596.49	4000.47
*m* = 72, *n* = 6	ALNS	640	7	1507.55	351.31	464.37	2970.23
	HALNS	640	0	1449.74	337.84	492.61	2920.19
pr18	AGA	1840	6	3639.82	848.21	1092.65	7426.68
*m* = 144, *n* = 4	ALNS	1200	7	2585.66	602.55	822.55	5217.77
	HALNS	1200	30	2561.08	596.82	765.11	5153.01

Note: m is the number of CDW generation sites, and n is the number of transfer stations.

## Data Availability

Not applicable.
